# Knowledge of Dengue Among Students Exposed to Various Awareness Campaigns in Model Schools of Islamabad: A Cross-Sectional Study

**DOI:** 10.7759/cureus.2455

**Published:** 2018-04-10

**Authors:** Nismat Javed, Haider Ghazanfar, Sajida Naseem

**Affiliations:** 1 Shifa College of Medicine, Shifa Tameer-E-Millat University Shifa International Hospital; 2 Internal Medicine, Shifa College of Medicine, Islamabad, PAK; 3 Department of Community and Family Medicine, Shifa International Hospital, Islamabad, Pakistan

**Keywords:** dengue, awareness campaigns, pakistan

## Abstract

Objective

To determine the knowledge of dengue among school students exposed to various awareness campaigns in model schools of Islamabad.

Methods

We conducted a cross-sectional study of students who were studying in Islamabad Model School for Girls F-7/2 and Islamabad Model College for Boys F-7/3 from September 2017 to October 2017. Students in the ninth and tenth grades who were willing to participate in the study and who were studying in the school for more than six months were included in the study. The data was collected through a self-constructed questionnaire. Cronbach's alpha was used to assess the internal consistency of the questionnaire, and it was found to be 0.83. The data obtained was analyzed on IBM's statistical package for the social sciences (SPSS) version 21 (IBM, Armonk, NY).

Results

Out of 601 participants, 345 (57.4%) were males and 256 (42.6%) were females. The mean age of the participants was 14.72±1.09. About 380 participants (63.2%) were studying in the ninth grade and 221 participants (36.8%) were studying in the tenth grade. A majority of the participants (67.2%) had poor knowledge of dengue. The participants scored highest in knowledge of prevention of the dengue domain and scored the lowest in knowledge of transmission of dengue. A majority of the participants (72.9%) reported that they acquire knowledge about dengue fever through television and radio. About 44.60% of the participants reported that they acquired knowledge about dengue fever through awareness campaigns in school.

Conclusions

The knowledge of the students was found to be insufficient despite several awareness campaigns. There is a need to re-evaluate the structure of the awareness campaigns as they fail to reach their target. Electronic media was identified as the most useful source of knowledge, and its incorporation can help increase the effectiveness of awareness campaigns.

## Introduction

Dengue is one of the diseases prevalent in the urban and semi-urban areas of Pakistan. According to World Health Organization (WHO), approximately half of the world's population is at risk of developing dengue. Around 390 million people develop dengue infections every year [1], and approximately 96 million people manifest with clinical symptoms of the infection. Another study done in 128 countries estimated that around 3.9 billion people got infected with dengue every year [2]. The number of cases has increased from 2.2 million in 2010 to 3.2 million in 2015. [1]. Dengue is now endemic in more than 100 countries. South-East Asia, America, and the Western Pacific regions are the most extremely affected regions.

Dengue virus (DENV) -1, -2, -3, and -4 are the four major serotypes of the virus [3]. The female mosquito species of Aedes aegypti is the major vector for the transmission of the dengue infection. This species of mosquito is a daytime feeder and its peak biting periods are the early morning hours and the evening hours before the dusk. An uninfected mosquito becomes infected when it bites an infected person. Symptomatic or asymptomatic humans are the main carriers of this virus.

Pakistan is one of the countries that have been severely affected by the dengue infection. According to Health Departments in Pakistan, around 23,000 people were admitted because of this infection in 2011 [4]. DENV-2 and DENV-3 were the two most common serotypes leading to the dengue outbreak in Punjab, Pakistan, in 2011 [5]. A person who recovers from infection by one serotype develops lifelong immunity to that specific serotype. Subsequent infection from another serotype can result in the development of “dengue hemorrhagic fever.”

Having sufficient knowledge of dengue is important in order to prevent the spread of this disease. Various awareness campaigns have been conducted all over Pakistan since the start of the dengue outbreak in Pakistan. Despite various awareness campaigns, the prevalence of dengue infection has not significantly decreased. The purpose of our study was to assess the knowledge gained by the schoolchildren after various awareness campaigns were conducted in their school.

## Materials and methods

We conducted a cross-sectional study on students who were studying in Islamabad Model School for Girls F-7/2 and Islamabad Model College for Boys F-7/3 from September 2017 to October 2017. Students of the ninth and tenth grades who were willing to participate in the study and who were studying in the school for more than six months were included in the study. Students who were not present on the days of the study were not included in the study.

The data collection was divided into two parts. The first part of data collection was the administration of a self-constructed questionnaire. It compromised of two sections. The first section contained questions pertaining to the demographic information of the participants while the second section contained questions assessing the knowledge of participants regarding dengue. The second section was further divided into four domains. The four domains are as follows: knowledge of the transmission of dengue, knowledge of the signs and symptoms of dengue, knowledge of the prevention of dengue, and the source of knowledge of dengue. Cronbach's alpha was used to assess the internal consistency of the questionnaire [6] and was found to be 0.83. The responses to the knowledge of the transmission of the dengue domain, knowledge of the signs and symptoms of the dengue domain, and knowledge of the prevention of the dengue domain were scored. Each correct answer was scored 1 and each wrong answer was scored 0. The "Do not know" response was considered a wrong answer and was scored 0. The maximum score for knowledge of the transmission of the dengue domain was 8 while the maximum score for both knowledge of the signs and symptoms of the dengue domain and knowledge of the prevention of the dengue domain was 9. The highest total score possible was 26. Participants who got a score of more than 80% were defined as having good knowledge of dengue fever; participants who got less than 60% were defined as having poor knowledge of dengue fever; while participants whose score was between 60% and 80% were defined as having average knowledge of dengue fever.

The questionnaire was distributed to all sections of the ninth and tenth grades. All the participants were given 20 minutes to complete the questionnaire. Consent was also taken from the administration of the participating institutions. A total of 663 questionnaires were distributed, out of which 601 questionnaires (90.6%) were filled. In the second part of data collection, a 10-minute interview was carried out to assess the in-depth knowledge of dengue. After the data collection, all the students were given a seminar on dengue. A question and answer session was held after the seminar to address any queries regarding dengue.

The data obtained was analyzed on IBM's statistical package for the social sciences (SPSS) version 21 (IBM, Armonk, NY). Descriptive statistics were used to analyze and describe the data. Frequencies and percentages were calculated for qualitative variables like gender and source of information about dengue. Mean and standard deviation (SD) were calculated for quantitative variables like age and domain scores. The Pearson chi-square test was applied to determine if there was any significant difference between gender and the source of information about dengue fever. The independent sample t-test was used to assess the association of knowledge regarding dengue with various variables. A p-value of less than < 0.05 was considered significant.

## Results

**Figure 1:**
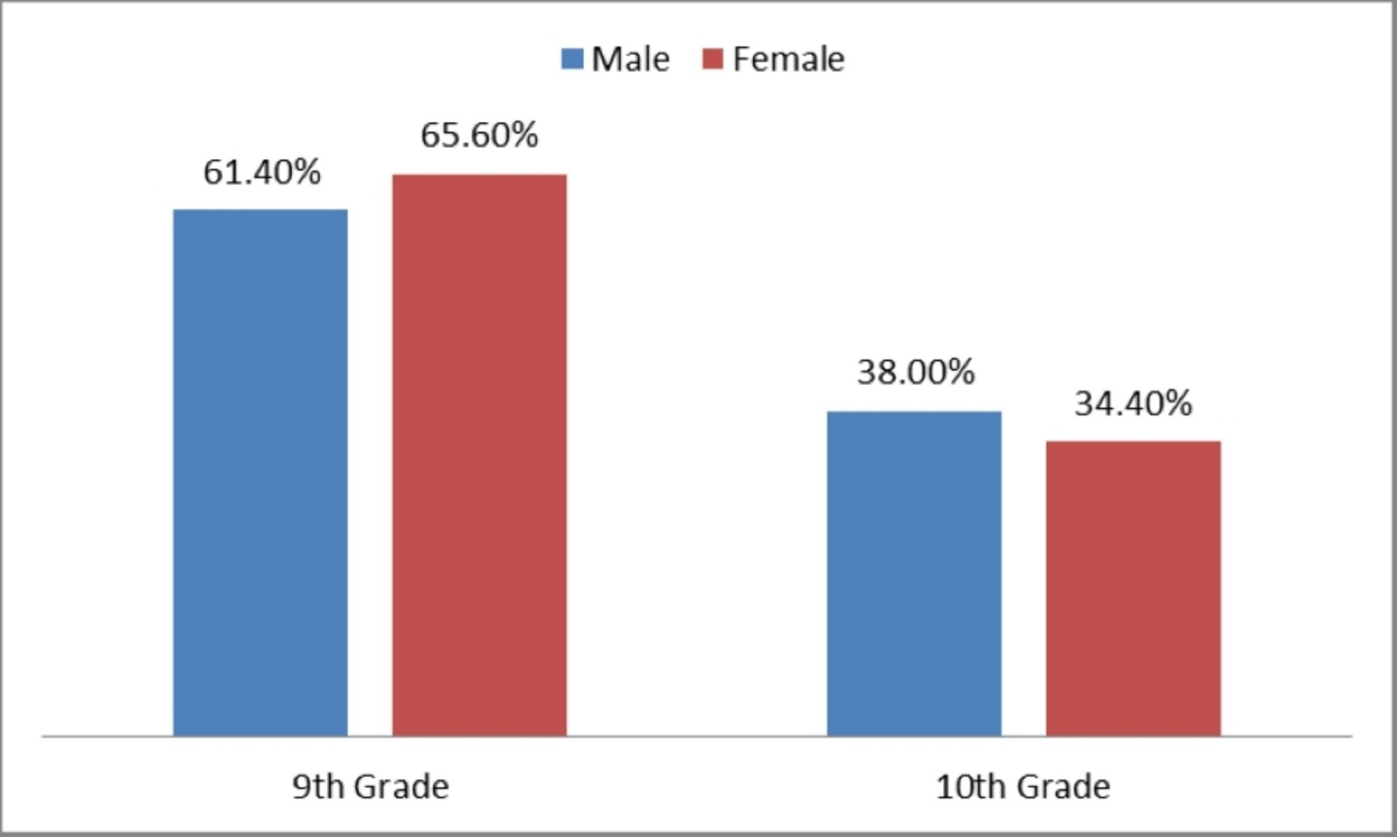
Distribution of the participants according to their gender and class

Out of 601 participants, 345 (57.4%) were males and 256 (42.6%) were females. The mean age of the participants was 14.72±1.09. About 380 participants (63.2%) were studying in the ninth grade and 221 participants (36.8%) were studying in the tenth grade. There was no significant difference between grade and gender (p-value=0.306).The distribution of male and female participants according to their grade has been presented in Figure 1.

Based on the criteria mentioned in the materials and methods section, only one participant (0.2%) had good knowledge of dengue while 32.8% participants had average knowledge of dengue and 67.1% participants had poor knowledge of dengue. Participants scored the lowest in knowledge of the transmission of the dengue domain and scored the highest in knowledge of the prevention of the dengue domain. This has been presented in Table 1.

**Table 1:** Domains score

Domains	Total Score
Knowledge of the transmission of dengue	4.13±1.40
Knowledge of the signs and symptoms of dengue	4.58±1.31
Knowledge of the prevention of dengue	5.59±1.54
Overall score	14.30±2.57

The independent t-test was applied to assess if there was any association between the gender of the participants and knowledge of the transmission of the dengue domain. P-value < 0.05 was considered significant. Male participants were found to have a significantly higher total score in this domain as compared to female participants. This has been presented in Table 2.

**Table 2:** Association between gender and score of knowledge of the transmission of the dengue domain p<0.05 was considered significant

Questions	Gender	Score	p-value
Which species of mosquito transmits dengue?	Male	0.374±0.48	<0.05
	Female	0.484±0.50	
Which is the age group most affected by dengue?	Male	0.620±0.48	< 0.001
	Female	0.429±0.49	
Do all mosquitoes transmit dengue?	Male	0.759±0.43	>0.05
	Female	0.801±0.39	
Do mosquitoes breed in standing/stagnant water?	Male	0.661±0.46	<0.001
	Female	0.621±0.49	
Does an uninfected mosquito become infected when it bites an infected person?	Male	0.327±0.47	>0.05
	Female	0.394±0.49	
Are dengue mosquitoes most likely to bite during the daytime?	Male	0.443±0.50	>0.05
	Female	0.449±0.50	
Is one most likely to get dengue during the breeding season of the dengue mosquito?	Male	0.264±0.44	>0.05
	Female	0.207±0.41	
Does ordinary person-to-person contact transmit dengue?	Male	0.707±0.45	>0.05
	Female	0.707±0.46	
Total score of knowledge of transmission of the dengue domain	Male	4.156±1.40	<0.05
	Female	4.102±1.40	

The independent t-test was applied to assess if there was any association between the gender of the participants and knowledge of the signs and symptom of the dengue domain. P-value < 0.05 was considered significant. Female participants were found to have a significantly higher total score in this domain as compared to male participants. This has been presented in Table 3.

**Table 3:** Association between gender and score of knowledge of the signs and symptoms of the dengue domain p<0.05 was considered significant

Questions	Gender	Score	p-value
Is fever a symptom of dengue fever?	Male	0.919±0.27	>0.05
	Female	0.922±0.27	
Is a headache a symptom of dengue fever?	Male	0.759±0.43	<0.05
	Female	0.828±0.38	
Is rash a symptom of dengue fever?	Male	0.496±0.50	<0.001
	Female	0.375±0.48	
Are vision problems a symptom of dengue fever?	Male	0.093±0.29	>0.05
	Female	0.098±0.30	
Is unconsciousness a symptom of dengue fever?	Male	0.101±0.30	>0.05
	Female	0.129±0.33	
Is vomiting a symptom of dengue fever?	Male	0.516±0.50	>0.05
	Female	0.594±0.49	
Is muscle pain a symptom of dengue fever?	Male	0.562±0.49	>0.05
	Female	0.605±0.49	
Is pain behind the eyes a symptom of dengue fever?	Male	0.649±0.48	>0.05
	Female	0.687±0.46	
Is bleeding a symptom of dengue fever?	Male	0.359±0.48	<0.001
	Female	0.508±0.50	
Total score of knowledge about the signs and symptoms of the dengue domain	Male	4.455±1.33	<0.001
	Female	4.761±1.26	

The independent t-test was applied to assess if there was any association between the gender of the participants and knowledge of the prevention of the dengue domain. P-value < 0.05 was considered significant. Although female participants had a higher total score in knowledge of the prevention of the dengue domain than male participants, it was not statistically significant (p > 0.05). This has been presented in Table 4.

**Table 4:** Association between gender and score of knowledge of the prevention of the dengue domain p<0.05 was considered as significant

Questions	Gender	Score	p-value
Use mosquito repellent/cream	Male	0.542±0.50	>0.05
	Female	0.566±0.49	
Eliminate standing/stagnant water to reduce mosquitoes	Male	0.661±0.47	>0.05
	Female	0.617±0.49	
Clean garbage	Male	0.649±0.48	>0.05
	Female	0.648±0.48	
Wear long sleeves and long pants to cover your arms and legs	Male	0.817±0.39	>0.05
	Female	0.812±0.39	
Closing doors and windows at biting time	Male	0.699±0.46	>0.05
	Female	0.718±0.45	
Restricting the time spent outside during the biting hours of the dengue mosquito	Male	0.278±0.45	>0.05
	Female	0.226±0.42	
Use mosquito nets and mosquito coils	Male	0.783±0.42	>0.05
	Female	0.793±0.42	
Turning containers and pots upside down to prevent water collection	Male	0.412±0.49	>0.05
	Female	0.449±0.50	
Use of insecticide sprays	Male	0.278±0.45	>0.05
	Female	0.336±0.47	
Total score of knowledge about the prevention of dengue	Male	5.539±1.61	>0.05
	Female	5.660±1.43	

**Figure 2:**
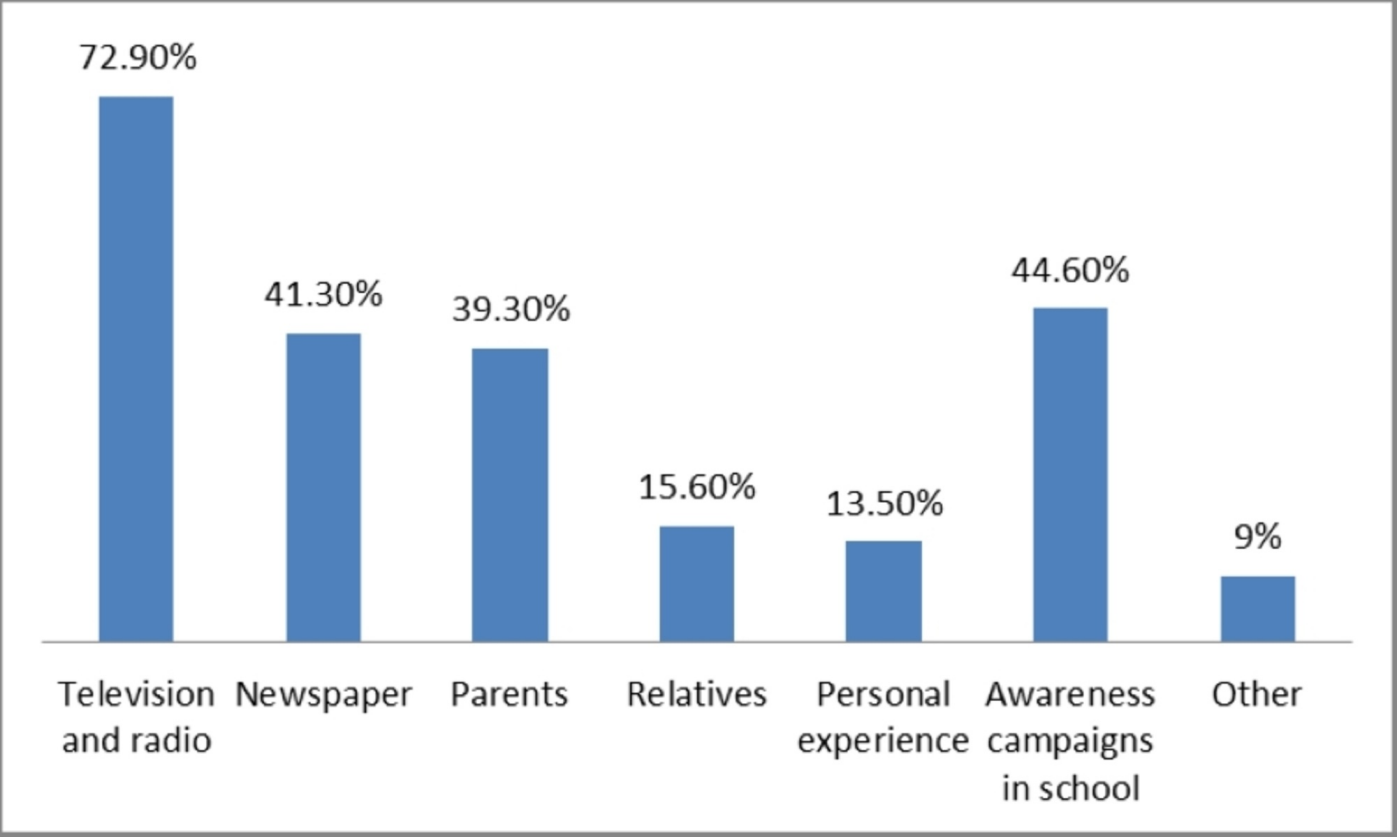
Source of information on dengue fever

A majority of the participants (72.9%) reported that they acquired knowledge of dengue fever through the television and radio. There was no significant difference between gender and source of information (p > 0.05). About 44.60% of the participants reported that they acquired knowledge of dengue fever through awareness campaigns in school. This has been presented in Figure 2.

## Discussion

There have been several outbreaks of dengue fever since the first case of dengue was reported in 1994 [7-8]. Various awareness campaigns have been carried out in Pakistan since the start of these outbreaks. There is a plethora of research articles assessing the effectiveness of these awareness campaigns. Despite various campaigns and preventive measures, dengue is still endemic in Pakistan. 

Our study shows that despite the various awareness campaigns in schools, a majority of the students had poor or average knowledge of dengue fever. A study carried out in Nepal concluded that only 12% of the participants had good knowledge of dengue [9] while a study done in Jamaica concluded that 87% of the participants had poor knowledge of dengue [10]. A study in Laos reported that 93% of the participant believed that they had insufficient knowledge of dengue [11].

A majority of the participants correctly identified fever and headache as the common symptoms of dengue fever.These were also reported as the common symptoms of dengue fever in studies conducted in Nepal [9] and Laos [11]. A majority of the participants scored poorly on questions related to the signs and symptoms pertaining to the complications of dengue. About 90.5% of the participants did not know that dengue fever can cause vision problems while only 42.3% of the participants reported bleeding from body orifices as a symptom of dengue. Although the ophthalmic complications of dengue are rare, they have been reported in several studies [12-13]. Having insufficient knowledge of the signs and symptoms of dengue can lead one to confuse dengue fever with other illnesses.

About 64.4% of the participants did not know that an uninfected mosquito can become infected when it bites an infected person and 29.3% of the participants thought that dengue can spread through direct contact with an infected person. There is a need to address these misconceptions. A study carried out in Jamaica also reported that only 40% of the participants were aware that person-to-person contact cannot spread dengue [14].

A majority of the participants (72.9%) reported that they acquired knowledge of dengue fever through television and radio. Several other studies also identified television and radio as the most common source of information on dengue fever [9,15]. Electronic media can play a significant role in conveying information too. 

As the transmission of dengue fever domain had the lowest score, there is a need to focus awareness campaigns on this aspect. Improving knowledge regarding transmission might also make it easy for participants to understand the reason for various preventive steps that have to be taken in order to prevent the transmission of dengue [16]. There is a dire need to re-evaluate awareness campaigns so that effective awareness campaigns can be carried out in the future. Workshops on dengue can also be organized by the school administration for teachers and students, to raise awareness about dengue.

## Conclusions

Student knowledge was found to be insufficient despite several awareness campaigns. There is a need to re-evaluate the structure of the awareness campaigns, as they fail to reach their target. Electronic media was identified as the most useful source of knowledge, and its incorporation can help increase the effectiveness of awareness campaigns.
